# Achieving health equity in immune disease: leveraging big data and artificial intelligence in an evolving health system landscape

**DOI:** 10.3389/fdata.2025.1621526

**Published:** 2025-11-14

**Authors:** Stan Kachnowski, Asif H. Khan, Shadé Floquet, Kendal K. Whitlock, Juan Pablo Wisnivesky, Daniel B. Neill, Irene Dankwa-Mullan, Gezzer Ortega, Moataz Daoud, Raza Zaheer, Maia Hightower, Paul Rowe

**Affiliations:** 1 Healthcare Innovation and Technology Lab, New York, NY, United States; 2 Columbia Business School, Columbia University, New York, NY, United States; 3 Sanofi, Morristown, NJ, United States; 4 Sanofi, Cambridge, MA, United States; 5 Walgreens Boots Alliance, New York, NY, United States; 6 Division of General Internal Medicine, Icahn School of Medicine at Mount Sinai, New York, NY, United States; 7 Division of Pulmonary, Critical Care, and Sleep Medicine, Icahn School of Medicine at Mount Sinai, New York, NY, United States; 8 Courant Institute of Mathematical Sciences, Department of Computer Science, New York University, New York, NY, United States; 9 Department of Health Policy and Management, Milken Institute School of Public Health, George Washington University, Washington, DC, United States; 10 Center for Surgery and Public Health, Department of Surgery, Brigham and Women's Hospital, Harvard Medical School, Boston, MA, United States; 11 Veritas Healthcare Insights, Park City, UT, United States

**Keywords:** health equity, AI, machine learning, big data, big data analytics, immunology, immune disease

## Abstract

Prevalence of immune diseases is rising, imposing burdens on patients, healthcare providers, and society. Addressing the future impact of immune diseases requires “big data” on global distribution/prevalence, patient demographics, risk factors, biomarkers, and prognosis to inform prevention, diagnosis, and treatment strategies. Big data offer promise by integrating diverse real-world data sources with artificial intelligence (AI) and big data analytics (BDA), yet cautious implementation is vital due to the potential to perpetuate and exacerbate biases. In this review, we outline some of the key challenges associated with achieving health equity through the use of big data, AI, and BDA in immune diseases and present potential solutions. For example, political/institutional will and stakeholder engagement are essential, requiring evidence of return on investment, a clear definition of success (including key metrics), and improved communication of unmet needs, disparities in treatments and outcomes, and the benefits of AI and BDA in achieving health equity. Broad representation and engagement are required to foster trust and inclusivity, involving patients and community organizations in study design, data collection, and decision-making processes. Enhancing technical capabilities and accountability with AI and BDA are also crucial to address data quality and diversity issues, ensuring datasets are of sufficient quality and representative of minoritized populations. Lastly, mitigating biases in AI and BDA is imperative, necessitating robust and iterative fairness assessments, continuous evaluation, and strong governance. Collaborative efforts to overcome these challenges are needed to leverage AI and BDA effectively, including an infrastructure for sharing harmonized big data, to advance health equity in immune diseases through transparent, fair, and impactful data-driven solutions.

## 1 Introduction

Prevalence and burden of immune diseases, including asthma, atopic dermatitis, rheumatoid arthritis (RA), multiple sclerosis (MS), and inflammatory bowel disease (IBD), are increasing in high-income countries, and recent estimates suggest a prevalence of approximately 1 in 10 individuals for immune diseases (Lerner et al., 2015; Cao et al., 2023; Conrad et al., 2023; Miller, 2023a; Shin et al., 2023; Wang et al., 2023). The rise in these often lifelong, progressive, and incurable immune diseases (Wylezinski et al., 2019) is alarming, and despite population growth playing a role, the underlying reasons are unclear. However, as immune diseases occur in genetically predisposed individuals following exposure to environmental factors (e.g., chemicals, dietary components, gut dysbiosis, and infections) (Vojdani et al., 2014; Pisetsky, 2023), it is likely that evolving environmental exposures may explain the increases in autoimmunity and immune disease (Miller, 2023a). As public health data collection and analysis over the past 5 decades has improved, environmental factors and occupational exposures have emerged that appear to be unevenly distributed across populations, as evidenced by the socioeconomic and regional disparities underpinning immune diseases (Quinn et al., 2007; Roberts and Erdei, 2020; Conrad et al., 2023; Global Burden of Disease (GBD) 2019 IMID Collaborators, 2023). For example, changes to these exposures/disparities may explain the increasing prevalence of MS among African Americans, particularly women, who have overtaken White individuals as the population at greatest risk (Goonesekera et al., 2024). Outcome disparities are also common in minoritized populations with immune disease and include underdiagnosis, suboptimal treatment, higher morbidity, worse quality of life, and higher mortality (Davis et al., 2021; Global Burden of Disease (GBD) 2019 IMID Collaborators, 2023).

To address the future impact of immune disease, data on the distribution, risk factors (genetic, behavioral, and environmental), and biomarkers have been proposed to enhance disease understanding, develop preventive strategies, and improve diagnosis and treatment (Lerner et al., 2015; Peng et al., 2021; Miller, 2023a). Such evidence can be obtained through “big data,” defined by the seven Vs (Volume, Velocity, Variety, Variability, Veracity, Visualization, and Value) (Batko and Slezak, 2022), that consolidate real-world clinical, research, biometric, patient-reported outcome, social, and financial data. Further, by collecting health, socioeconomic, and sociodemographic data, big data have the potential to improve understanding of health disparities and identify approaches to improve health equity (Galea and Abdalla, 2023). A necessity of big data, owing to its complexity and unstructured sources, is the use of artificial intelligence (AI)-powered big data analytics (BDA), such as machine learning (ML), whereby computers use algorithms to learn from data and improve task performance (e.g., prediction of outcome variables) (Fuller et al., 2017). BDA in healthcare comprises data collection, storage, analysis, data mining, and ML techniques to provide descriptive, predictive, prescriptive, and discovery analytics using large volumes of omics, biomedical, telemedicine, and electronic health record (EHR) data, enabling big data to inform preventive and precision medicine (Bartoloni et al., 2022; Batko and Slezak, 2022).

Although AI and BDA can facilitate the identification and resolution of health inequities (Galea and Abdalla, 2023), and has been used extensively in immune disease to facilitate early diagnosis or prognostic models (Danieli et al., 2024), it can perpetuate inequities if the data are not representative of minoritized populations (Norori et al., 2021; Gurevich et al., 2023). For example, unrepresentative training data or other flawed/biased assumptions may result in algorithmic bias (Gurevich et al., 2023), whereby existing inequities are compounded or amplified by algorithms that erroneously assign patients with different needs, or levels of risk, with the same algorithm score (or vice versa) (Obermeyer et al., 2019; Panch et al., 2019; Chin et al., 2023). As AI and BDA have applications across the full spectrum of healthcare (diagnosis and treatment, prognosis/risk stratification, triage, and resource allocation), there is potential of various levels of benefit and harm (Favaretto et al., 2019; Peng et al., 2021; Batko and Slezak, 2022; Chin et al., 2023; Gurevich et al., 2023; Danieli et al., 2024). The causes of discrimination in data analytics, solutions to discrimination in big data, and barriers to their adoption have been reviewed previously (Favaretto et al., 2019). Additionally, various frameworks and interdisciplinary approaches have been proposed to ensure AI and BDA promote, and do not hinder, health equity (Ibrahim et al., 2020; Clark et al., 2021; Dankwa-Mullan and Weeraratne, 2022; Chin et al., 2023). This review outlines some of the key challenges associated with achieving health equity in immune diseases through the use of big data, AI, and BDA, together with potential solutions.

## 2 Challenges and solutions in implementing big data to address health equity

**Figure 1:**
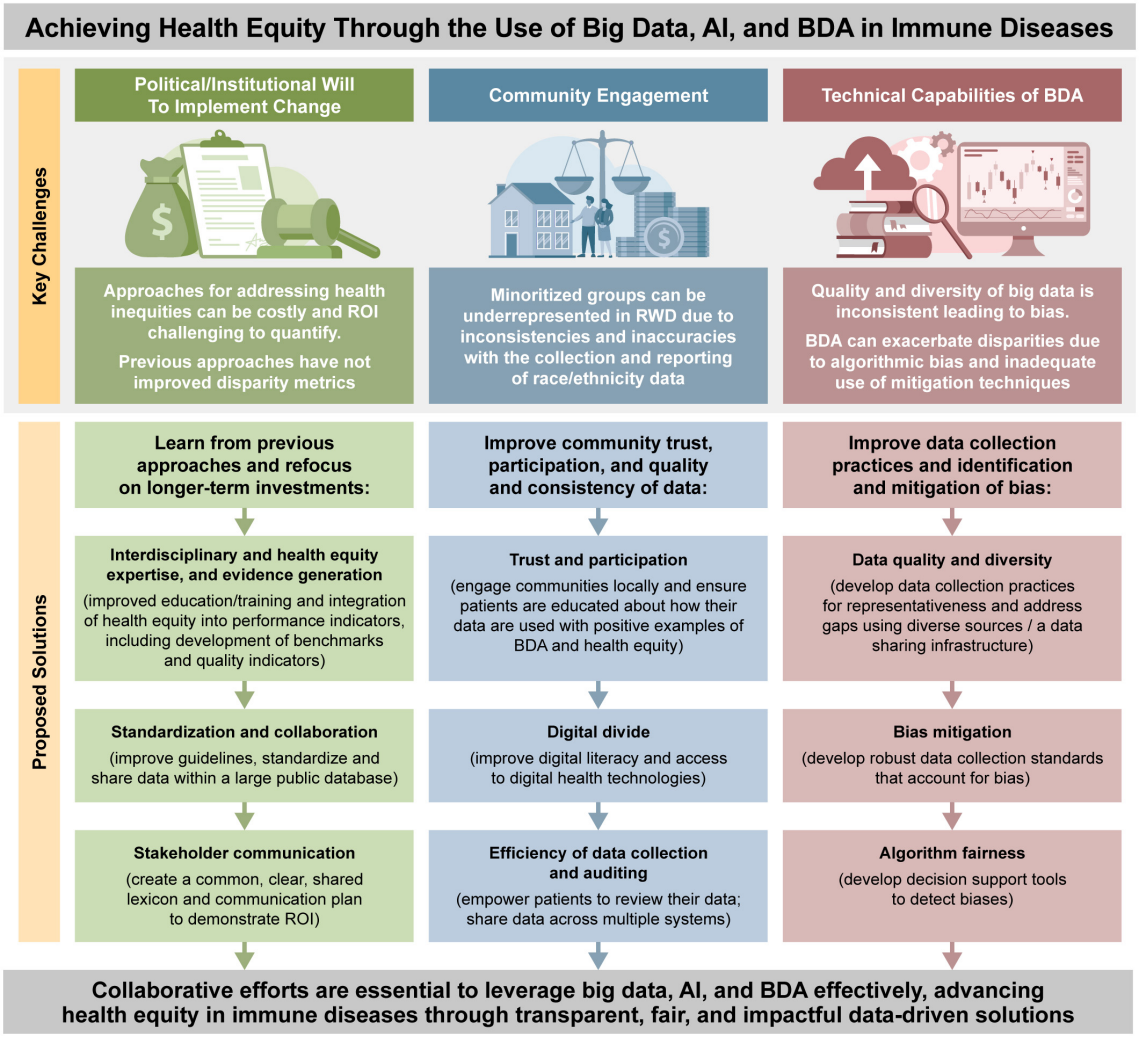
Overview of key challenges and proposed solutions to achieve health equity through use of big data and BDA in immune diseases. AI, artificial intelligence; BDA, big data analytics; ROI, return on investment, RWD, real-world data.

As highlighted by a recent systematic literature review, there are underlying challenges to the implementation of AI and BDA within immunology and allergy, including poor data quality and quantity, limited access to shared datasets, geographic bias, the high resource burden of managing complex data, lack of AI model interpretability, inadequate clinician training on AI integration, and ethical concerns around privacy, bias, and regulation (Xiao et al., 2025). We will discuss these issues in relation to health equity, aiming to identify solutions to key challenges such as political/institutional will to implement change [e.g., evidence to support a return on investment (ROI)], community engagement, and technical capabilities of AI and BDA (Galea and Abdalla, 2023) (see Figure 1).

### 2.1 Political/institutional will to implement change: challenges and solutions to a lack of interdisciplinary subject-matter experts

The political/institutional will to implement change can be defined as obtaining buy-in from key decision makers, along with the commitment and capacity of healthcare organizations, research institutions, and industry stakeholders to drive meaningful improvements in health equity. Indeed, health institutions play a critical role in either perpetuating or addressing health inequities and must be held accountable for their impact (Chisolm et al., 2023). Given the complexities introduced by shifting political administrations and congressional policies on health equity initiatives, these healthcare institutions are uniquely positioned to drive systemic change (Deloitte Center for Health Solutions, 2025).

#### 2.1.1 Increasing awareness of health equity initiatives to foster multidisciplinary expertise

The key to driving political/institutional and wider systemic change needed to address health equity in immune disease is improving evidence-generation and its communication by subject-matter experts of the benefits of AI and BDA to key stakeholders (e.g., payers, clinicians, and regulators). While there are subject-matter experts in AI/BDA, health equity, or immune diseases, there is understandably a limited pool of experts proficient in all these areas. The benefits of AI and BDA therefore need to be communicated more widely, particularly in relation to health equity, to ensure it is widely adopted and these multidisciplinary experts can be fostered.

One approach could be to increase awareness at educational institutions and offer multidisciplinary undergraduate/postgraduate training of immunologists regarding computational biology, programming, and bioinformatics, among other BDA-related topics (Schultze, 2015). Creation of a common, clear, and shared lexicon, built on existing information (Fuller et al., 2017; American Medical Association and Association of American Medical Colleges, 2021), will also be important to minimize inconsistencies and facilitate the synthesis and comparison of data (Palaniappan et al., 2024) so that the benefits of AI and BDA for health equity can be understood. These definitions could be incorporated into data guidelines and standards to inform multidisciplinary consortia involved in decision-making. For example, the Findable, Accessible, Interoperable, Reusable (FAIR) data principles offer domain-independent guidelines for producers and publishers to enhance data reusability through effective data management and stewardship (Wilkinson et al., 2016). More recently, the Gravity Project has provided consensus-based standards on how social determinants of health (SDoH) data are used and shared (Gravity Project, 2025). In addition to establishing a clear lexicon aligned with existing data guidelines and standards, it is important to define trackable key performance indicators (KPIs) to assess the value of health equity initiatives. Multidisciplinary expertise should be engaged to develop a communication strategy for health equity in big data/BDA, effectively conveying these KPIs and highlighting areas with potential or demonstrated ROI.

#### 2.1.2 Utilizing KPIs to communicate the value of health equity initiatives

Evaluating the role of AI, ML, deep learning, and advanced analytics in promoting health equity for immune disorders requires a multidimensional set of KPIs that must address data inclusivity, diagnostic fairness, patient outcomes, and real-world implementation, with a persistent focus on addressing disparities rather than just improving averages. To ensure KPIs are communicated to and used effectively by key stakeholders they could be incorporated into data guidelines, standards, and recommendations [e.g., FUTURE-AI (Lekadir et al., 2025)] and disseminated via a new position paper. Example KPIs for determining the success of big data efforts to achieve health equity are summarized in Table 1. A more exhaustive list is available in a white paper from the National Committee for Quality Assurance (Harrington et al., 2021).

**Table 1:** Overview of KPIs for determining the success of AI and BDA in promoting health equity.

**Category and example KPI**	**Contribution**
**Summary indices**	
**Health equity metric:** Zimmerman and Anderson (2019) Composite measure of health inequality, health disparities, and mean health decurved by the weighted deviation of individual health from the best achievable health of the privileged group	Offers a composite and sensitive measure capturing multiple dimensions of health disparities to provide a more nuanced understanding of health equity trends than single-outcome measures
**Health equity summary score:** Agniel et al. (2021) Composite score, based on clinical and patient experience, evaluating care quality for at-risk groups by measuring current performance, within-plan improvement, and progress against national equity benchmarks	Incentivizes health plans to reduce disparities and improve care for socially at-risk populations
**Outcome equity**	
**Health Equity Assessment of machine Learning performance (HEAL) metric:** Schaekermann et al. (2024) Investigates the likelihood that the AI model performs better for subpopulations with worse outcomes than others (outcomes include DALYs and YLLs)	The HEAL metric helps to determine whether AI tools are capable of prioritizing disadvantaged patients with worse outcomes, thus helping to improve health equity
**Equity sensitive QALYs:** Lindemark et al. (2014) Uses lifetime QALYs and proportional shortfall to identify who is “worse off” by measuring total and relative health deficits before intervention	By prioritizing health gains for those with fewer lifetime QALYs, the method enables equity-aware resource allocation
**Fairness metrics**	
**Group fairness:** Mienye et al. (2024) Determines whether predictive outcomes are independent of sensitive attributes including race, gender, and age	Ensures that the probability of a positive outcome is the same across different groups, aiming to equalize a statistical measure (e.g., PPV) across sensitive attributes
**Performance by demographics** (Rajkomar et al., 2018)	
**Equal outcomes:** ensures the ML model results in equal benefit for protected and non-protected groups **Equal performance:** ensures model performance/accuracy is similar for the protected and non-protected groups by determining model sensitivity (equal opportunity), sensitivity and specificity (equalized odds), and positive predictive value **Equal allocation:** ensures resources are proportionately allocated to the protected group	Determines if outcome, performance, and allocation metrics are similar for protected and non-protected groups. The ML model can be refined to ensure equal outcomes for patients; however, care is required because a model can be fair for some metrics and not others, requiring clinical and ethical reasoning to determine the appropriateness of the data
**Explainability and trust**	
Six metrics to quantify the extent to which model predictions can be explained: Munoz et al. (2023) **Importance concentration** (feature importance spread, alpha-feature importance), **importance consistency** (rank assignment, rank consistency, importance stability), **prediction fluctuation** (fluctuation ratio, performance degradation), **surrogate fidelity** (surrogate fidelity score, performance degradation), **surrogate stability** (surrogate feature stability), **global surrogate performance** (global surrogate model performance, e.g., R^2^ or accuracy)	Provides a systematic and interpretable framework for evaluating AI models by assessing multiple aspects of model complexity and explainability, enabling a comprehensive understanding of how models generate their outputs
The Galileo human evaluation framework uses conceptual **human evaluation metrics** such as correctness, completeness, and context adherence, which are typically scored on Likert scales, to capture nuanced human judgments of AI output quality (Bronsdon, 2025)	By engaging diverse populations, these metrics can help ensure AI outputs are accurate, relevant, and inclusive supporting more equitable health communication and decision-making
**Financial return**	
**Social return on investment:** Ashton et al. (2024) Assigns financial proxy values to non-financial outcomes, making intangible social impacts more visible by evidencing the broader social value generated from financial investments. A ratio is developed based on social value in financial terms for every £1 invested	By capturing perspectives from multiple stakeholders, SROI can help quantify the broader benefits of public health interventions

AI, artificial intelligence; BDA, big data analytics; DALY, disability-adjusted life year; KPI, key performance indicator; ML, machine learning; PPV, positive predictive value; QALY, quality-adjusted life year; SROI, social return on investment; YLL, years of life lost.

In terms of BDA use more broadly, KPIs need to demonstrate minimized deviation from a gold-standard level of representativeness, minimized outcome disparity, and most importantly usability and scalability within robust healthcare systems. As proposed by Zimmerman and Anderson (2019), multiple metrics for health equity (health inequality, health disparities, and mean health) could be consolidated using an approach based on deviations from the best achievable health, defined by the median experience of the most privileged group (Zimmerman and Anderson, 2019). In addition to the traditional financial ROI metric, (net benefit – cost)/cost), an emerging concept is the creation of a single, blended metric that expands ROI beyond purely financial returns to include concepts such as value and social return on investment (SROI). SROI assigns financial proxy values to non-financial outcomes, making intangible social impacts apparent by demonstrating the broader social value generated from financial investments. Although SROI is recognized by domain experts as a valuable tool for demonstrating social value, awareness across the broader public health field remains limited. There is also a need for standardized methodologies and data reporting practices to ensure its validity and interpretability (Ashton et al., 2024).

### 2.2 Political/institutional will to implement change: challenges and solutions to stakeholder buy-in and maintaining investment

#### 2.2.1 Learning from past failures and demonstrating ROI with health equity initiatives

Addressing health inequities in immune diseases can be costly and it is challenging to quantify a ROI. However, the unmet need is evident. Immune diseases are lifelong and expensive to treat, with direct costs in the US projected to be around $200–300 billion annually (Wylezinski et al., 2019; Miller, 2023b). Further, the estimated costs of health inequities in 2018, based on medical care expenditures, lost productivity, and costs associated with premature death, were approximately $450 billion for minoritized populations in the US (LaVeist et al., 2023). Considering the contribution of health inequities to disease costs, a recent study has demonstrated that there is great potential for ROI (Yerramilli et al., 2024), particularly in the light of well-documented impacts of SDoH (Braveman and Gottlieb, 2014) and the associated gains in health and productivity (Yerramilli et al., 2024). Despite the potential for strategies targeting SDoH to improve health outcomes and generate cost savings, literature on ROI is scarce (Nikpay et al., 2024). In the last 20 years the US government, non-governmental organizations, and corporations have invested over $179 billion in health equity (Aluko et al., 2023) and despite this—according to one independent analysis—many disparity metrics have shown little or no improvement (Zimmerman and Anderson, 2019). Aluko et al. proposed multiple reasons for the failure of previous health equity investments/approaches, including insufficient governance (see Section 2.6), limited workforce and capacity skill sets, and unsupportive data and technology infrastructure. Some of the proposed solutions from the authors included a refocus on longer-term commitments, and investment in BDA platforms capable of understanding, targeting, and tracking disparities over time (Aluko et al., 2023).

By integrating healthcare data from diverse sources, BDA has the potential to enhance clinical decision-support tools and aid development of personalized or population-based services (Schulte and Bohnet-Joschko, 2022). Indeed, big data has impacted patient care for decades by helping insurance companies incentivize preventive care, ultimately leading to reduced acute care costs and improved care equity (Sabet et al., 2023). A scoping review found that three-quarters of papers reporting economic evaluations of BDA for clinical decision-making corroborated expectations of cost savings, ranging from US$126 per patient to over US$500 million for the entire US healthcare system; however, the interpretation of results was limited by a lack of full and properly performed economic evaluations (Bakker et al., 2020). Even in the absence of robust economic/ROI data, investment in BDA remains attractive and has recently been supported by the US Department of Health and Human Services (HHS), who in 2022 pledged US$90 million to identify and reduce health disparities using new data-driven solutions (Sabet et al., 2023). For investment to continue it will be important for the HHS and other institutions to recurrently evaluate their initiatives, using appropriate KPIs to determine value and ROI. In the absence of large long-term cases, smaller initiatives, such as the EHR-enabled rheumatology registry developed by the American College of Rheumatologists (Gilvaz and Reginato, 2023), may offer the best opportunities to highlight the potential of BDA on health equity and ROI in the near term. Alternatively, researchers may be able to investigate potential for AI-powered insights into health inequalities by registering for established precision medicine initiatives such as the National Institutes of Health-funded “All of US Research Hub” that is built on strong privacy and trust principles including governance, transparency, consent, and data quality (All of Us Research Program, 2025). While funding for this program is declining, opportunities remain for funded partnerships that may help identify solutions to health inequalities (All of Us Research Program, 2025); however, such opportunities are limited and without clear evidence of ROI, competing institutional priorities (e.g., financial sustainability, regulatory compliance, or short-term efficiency gains) may take precedence, to the detriment of health equity.

#### 2.2.2 Integration and tracking of health equity performance indicators

A survey of US healthcare executives found only 36% have a specific budget dedicated to advancing health equity (Accenture, 2022). A more recent survey found that 43% of life sciences executives and 48% of healthcare executives found it challenging to incorporate health equity into their strategic, financial, and operational processes (Deloitte Center for Health Solutions, 2025). Even when institutions commit to implementing big data and health equity initiatives, they often lack the governance structures, mechanisms, and metrics to track and encourage delivery. This is because unlike regulatory compliance, which is tied to financial or legal consequences, health equity efforts driven by big data often remain voluntary and lack clear metrics for accountability. In this regard, the KPIs described in Table 1 could prove valuable for tracking progress against key health equity metrics, facilitating accountability, and assessing the success of related initiatives.

According to one survey, more than 40% of life sciences and healthcare executives had difficulties tracking the progress of health equity initiatives (Deloitte Center for Health Solutions, 2025). Furthermore, 32% of health equity leaders had no data on the impact of health equity initiatives on their organizations' financial indicators. The same survey reported that while health equity leaders have the potential to differentiate between short- and long-term goals, relatively few are involved in decisions related to technology and IT (14%) and use of AI (12%). These findings suggest that the economic models of healthcare delivery and biomedical research may not align with the investments (and ROI) needed to use big data for equity-focused interventions. However, this may change in the future if health equity leaders can identify the factors associated with cost savings and ROI or shift to incorporating SROI and other broader value-based assessments.

Health equity leaders, in particular, are needed to direct policy and investment opportunities. Additionally, considering the lack of performance incentives and the need to incentivize health equity in resource-limited providers, the Agency for Healthcare Research and Quality's stakeholder engagement recommended the development of equity-focused evidence-based quality indicators, use of federal data to develop health equity benchmarks, and development of toolkits to assist healthcare organizations with integrating health equity metrics into their performance management (Chisolm et al., 2023). A standardized set of health equity measures would enable value-based incentive programs to reward strategies that reduce performance gaps by addressing the unique challenges faced by disadvantaged populations, rather than assuming that improvements in overall population outcomes will automatically benefit at-risk groups (Chisolm et al., 2023). Looking ahead, further investigation is needed to understand which incentives have the greatest impact, as well as which groups of stakeholders are best positioned to deliver health equity improvements. A tiered approach to performance incentives has also been proposed to ensure efforts that fall short of key benchmarks are still recognized as progress (Chisolm et al., 2023).

#### 2.2.3 Investment in long-term projects, including big data and AI-powered BDA

As proposed by Aluko et al. (2023) investment in longer-term commitments, such as BDA, will be important to ensure the success of health equity initiatives. However, investment in AI-powered BDA platforms capable of tracking disparities over time is challenging owing to difficulties in collecting sufficient, reliable, and up-to-date information on health disparities. For example, understanding health disparities requires careful consideration of confounding factors, such as healthcare insurance in the US, and the selection of appropriate research questions and populations. To address the unmet need for frequent and granular data collection, particularly regarding SDoH, Sabet et al. discussed the potential benefits of a large national public database of anonymized patient data capable of collecting diverse metrics based on equitable data collection strategies (Sabet et al., 2023). To ensure that the database captures data from marginalized populations, these groups should be included in the process from the early design stages, the design should be adapted for those with low literacy or limited technological proficiency, and investment should be made in technology infrastructure and staff training to prepare for comprehensive data collection (Sabet et al., 2023). Further, recommendations and guidelines are needed to progress the field in an ethical and collaborative manner to ensure data collection and storage methodologies adhere to ethics regulations and data privacy laws, and that findings can be effectively translated into clinical decision-making (Gossec et al., 2020).

To address these challenges, several data standards and principles have been developed, such as the Clinical Data Interchange Standards Consortium and the FAIR Guiding Principles for Scientific Data Management and Stewardship (Wilkinson et al., 2016; cdisc, 2025). As mentioned previously, success of such a database would be predicated on the development and achievement of predefined KPIs (see Table 1). It would also need sufficient data to address the lack of information on rare immune diseases, which would benefit from consolidating information from multiple sources (Peng et al., 2021). A holistic approach to health equity remains difficult due to the fragmentation of patient data across EHR systems, insurance databases, and research cohorts, hindering the development of comprehensive, equity-driven insights. Without institutional commitment to data sharing, achieving health equity will be challenging. Promoting cross-sector collaboration and using data dashboards to deliver insights to researchers and policymakers could be one solution to expedite investment (Sabet et al., 2023) in big-data platforms for immune disease.

### 2.3 Community engagement: challenges and solutions to data collection

Improving minority group participation is key to ensuring AI and BDA can be utilized to further health equity. Community engagement is key and will ensure minoritized communities with similar socioeconomic status (SES) collaborate with healthcare providers in addressing issues affecting their wellbeing. While complex factors underlie the lack of inclusion of minoritized populations in clinical research (Bibbins-Domingo and Helman, 2022a; Turner et al., 2022), big data has the potential to address these; however, representative data is often lacking. For example, race and ethnicity data are inconsistently recorded in real-world data (RWD)—in one US-based study, as many as 30% of individuals' claims/EHR data had missing race/ethnicity information (Goonesekera et al., 2024). Additionally, an analysis by the UK Office of National Statistics (ONS) found differences in ethnicity data recording between EHR data and the UK census, highlighting consistency issues (Drummond, 2023). Following a desk review of the ONS data, it was found that patient ethnicity data were being incorrectly recorded due to subjective interpretation by medical staff, non-standardized ethnicity response options across healthcare settings, and data quality checks focused on completeness vs. accuracy (Drummond, 2023).

#### 2.3.1 Improving participation by fostering data ownership

A potential solution to these issues is to enhance data accuracy by increasing patient ownership, allowing patients to review, edit, or validate their personal information. In the global shift toward paperless healthcare, patient data are increasingly accessible through online portals and mobile applications (e.g., MyChart). These platforms typically incorporate multiple features that have been shown to encourage patient ownership, including multilingual support, consolidation of data across multiple connected systems to prevent inefficiencies (e.g., entry of similar data across multiple platforms), and protection of confidentiality (Peng et al., 2021; Vishwanatha et al., 2023). However, there are potential limitations—while these platforms may allow patients to view their data, they can lack functionality to directly edit or correct inaccuracies related to ethnicity. Additionally, they may introduce errors due to a limited set of standardized ethnicity response options. Feedback from service users and advocacy groups could help refine these systems; however, a global framework for standardizing ethnicity categories may be needed to support future data integration and better identify disparities.

A key concern with engaging patients in data collection is that access to digital health technologies (DHTs) and overall digital literacy, which are key digital determinants of health, can create a digital divide that impacts the representativeness of big data (Ibrahim et al., 2020; Eruchalu et al., 2021; Campanozzi et al., 2023; Chidambaram et al., 2024) and may affect efforts to increase the diversity and accuracy of patient-reported data. For example, despite DHTs being increasingly used by patients and physicians in the management of asthma, their usage in smartphone applications has been shown to widen the digital divide by SES, as not all individuals own smartphones (Kaplan et al., 2023). While such technologies can be used to facilitate earlier diagnosis of asthma (Al Meslamani, 2023), and also atopic dermatitis (Yanagisawa et al., 2023), there is potential for outcome disparities to arise due to earlier diagnosis/DHT use and treatment in groups of higher vs. lower SES. It is therefore important to engage communities, implement strong governance, and enhance public digital literacy to ensure that the digital divide is minimized rather than widened by the adoption of DHTs (Fernandes et al., 2024).

#### 2.3.2 Effectively and transparently communicating the unmet need and potential of AI and BDA

In addition to providing ownership and an infrastructure for patients to validate their data, the AIM-AHEAD (Artificial Intelligence/Machine Learning Consortium to Advance Health Equity and Researcher Diversity) US-based stakeholder listening sessions identified the need to engage communities locally, obtain buy-in for each population, and ensure algorithms are transparent and easily understood (Vishwanatha et al., 2023). Transparency in practices could go a long way—especially in community engagement—toward building capacity and readiness among those who industry needs as volunteers in medical product development. To facilitate this, patients should be provided with multilingual educational materials on how their data are used, who has access, and the short- and long-term benefits of participation and data sharing to optimize BDA outputs and health equity. Short-term benefits include improved patient trust and generalizability of clinical findings. Long-term benefits include greater innovation, improved access to effective medical interventions, reduced health disparities, and lower economic costs (Bibbins-Domingo and Helman, 2022b). This education could also highlight the different disease prevalences among different racial and ethnic groups for relevant immune diseases (Goonesekera et al., 2024), and the aims of health equity to ensure equitable access to care and outcomes. In parallel with initiatives aimed at improving patient engagement and reducing barriers to clinical trial participation, frameworks such as the Clinical trial Diversity Rating should be used to ensure that key stakeholders and regulatory bodies have the data and oversight needed to address remaining challenges (Agboola and Wright, 2024).

With the advent of natural language processing, AI can help improve the quality of patient educational materials by allowing near instant translation across multiple languages and by simplifying content to improve quality and readability, maintain or improve understandability, and improve actionability (Saatçi et al., 2024; Will et al., 2025). Table 2 presents a selection of case studies on the application of AI and BDA in immunology; however, while they have the potential to improve health equity, no evaluations were conducted—highlighting the need to track equity-related KPIs in future studies. For example, an ML model scouring EHR data for immune-driven traits has been used to identify patients in need of further testing—potentially accelerating diagnosis and treatment (Forrest et al., 2023), and achieving cost savings with earlier diagnoses (Wylezinski et al., 2019). This model also identified a high-risk subgroup that would likely be underdiagnosed based on a lack of testing (Forrest et al., 2023), which is especially useful given the high prevalence of misdiagnoses in immune diseases (Goonesekera et al., 2024). Additional positive examples of AI and BDA being applied to increase health equity in immune diseases, together with appropriate ways of assessing how effective the initiatives have been, would be helpful to increase community engagement.

**Table 2:** Case studies of AI and BDA in immunology to increase health equity.

**Case study type**	**Case study details**	**Implications**
Disease risk (Forrest et al., 2023)	An ML model was developed to predict the necessity for autoimmune disease testing by analyzing longitudinal EHR data from 161,584 individuals. The model demonstrated high accuracy in identifying patients who should undergo rheumatological evaluation	Allowed for earlier detection of the need for autoantibody testing and rheumatology encounters, identifying at-risk patients up to 5 years before traditional clinical assessments would typically do so, thereby potentially accelerating diagnosis for underserved patients
Disease progression (Wang et al., 2025)	AI was used to develop a risk score, based on real-world biobank data, to predict progression of RA and systemic lupus erythematosus from preclinical to disease-state stages	Potential to facilitate earlier diagnosis, treatment, and intervention. If applied to bigger and more diverse datasets, it may help improve outcomes for patients with health disparities
Precision medicine (Chen et al., 2019)	A deep learning model was trained on peptide–HLA binding data to predict HLA class II antigen presentation, enabling individualized insights for vaccine design and autoimmune disease risk assessment	Enabled early identification of immunogenic peptides across diverse HLA profiles, supporting development of individualized immunotherapies
Precision medicine (Wang et al., 2024)	The EXPRESSO AI algorithm was developed to understand complex trait risk genes associated with various autoimmune diseases and then used this information alongside a drug repurposing pipeline (CADRE) to identify potential therapeutics	The study identified multiple new drugs with therapeutic potential. If this approach is applied across diverse populations, it could reveal population-specific genetic variants, supporting the development of more equitable precision medicine
Treatment response (Yoosuf et al., 2022)	Multi-omics ML framework integrating baseline transcriptomic, proteomic, and flow cytometry data from female RA patients before initiation of anti-TNF therapy. The ML model predicted with significant accuracy which patients would respond to treatment	Enabled early identification of likely responders and non-responders, potentially guiding more personalized treatment decisions

AI, artificial intelligence; BDA, big data analytics; CADRE, cell type aware drug repurposing pipeline; EHR, electronic health record; EXPRESSO, EXpression PREdiction with Summary Statistics Only; HLA, human leukocyte antigen; ML, machine learning; RA, rheumatoid arthritis; TNF, tumor necrosis factor.

### 2.4 Technical capabilities of AI and BDA: challenges and solutions to data quality and diversity

While big data and BDA may be central to addressing health disparities and providing ROI for stakeholders, inconsistency in the quality and diversity of RWD is a key limitation. Incomplete data is, however, an inherent feature of RWD, which is usually unstructured and unlabeled. Further, as outlined above, data can be missing for minoritized populations, hindering data training and the interpretability and generalizability of findings (Peng et al., 2021). For example, ML models predicting asthma exacerbations in children showed greater algorithmic bias for low-SES populations due to more incomplete EHR data (Juhn et al., 2022).

Models require rigorous testing across diverse populations and settings; otherwise, they might perform well on one group but fail when applied to a different population due to overfitting and/or lack of external validation (Peng et al., 2021). This is of concern in immune diseases that are more prevalent in low-SES populations, such as systemic lupus erythematosus (SLE) (Conrad et al., 2023), and have complex genetic and environmental triggers (Vojdani, 2014; Pisetsky, 2023) that may impact minoritized communities to a greater extent. For example, the increased risk of RA and SLE in patients from low-SES groups (Conrad et al., 2023) and hypothesized genetic differences that may explain the earlier onset of immune diseases, including IBD, MS, RA, and SLE in minoritized populations (Sharma-Oates et al., 2022). While large, representative training datasets can address these issues, as shown by EHR-trained ML diagnostic models for RA and SLE (Forrest et al., 2023), and the EXPRESSO AI model identifying causal genes and potential immune disease-modifying compounds (Wang et al., 2024), minoritized groups remain underrepresented in genome-wide association studies (GWAS) for MS (Jacobs et al., 2022). Data gaps like this may perpetuate inequities and limit the potential of AI and BDA to inform personalized genomic medicine. For example, the lack of representativeness in GWAS may explain why only ~50% of the estimated heritability is currently understood for MS, which is diagnosed and treated earlier in people of European vs. non-European ancestry (Jacobs et al., 2022).

#### 2.4.1 Improving data quality with robust data collection and data harmonization

While community engagement is essential to increase the diversity of data and ensure the damaging effects of bias can be identified and mitigated, robust data collection standards are needed to account for bias and employ tools that address physician biases in diagnosis and measurement of patient outcomes. To expand and plug information gaps, it is essential to collect race, ethnicity, sex, gender, and other social risk factor data from diverse sources and educate stakeholders on the importance of maintaining accurate and complete records. Reviewing supplier data procurement contracts and incorporating bias-handling clauses may help ensure that disparities are actively mitigated. To facilitate improved data quality, a steering committee or leadership structure could be established by thought leaders or a governmental organization, such as the HSS. This committee would inform data collection practices and create a gold standard for representativeness, enabling the assessment of underrepresented intersectional subpopulations within big data. Collaboration is also crucial for improving access to healthcare data and sharing it within diverse communities; there is enormous potential for sharing data within a nationwide database (Sabet et al., 2023).

To ensure RWD from multiple sources (e.g., claims, Centers for Medicare and Medicaid Services, EHR, and demographic data) are useful, it will be important to harmonize and standardize the information by implementing paperless systems, standardizing metrics, and building an infrastructure for sharing data. Universal standards, such as Health Level Seven—Fast Healthcare Interoperability Resources (HL7 FHIR), should be applied to provide a standardized way of formatting and exchanging healthcare data, making it easier for different systems (e.g., EHRs, apps, hospitals, and insurers) to communicate and share data consistently and securely (HL7 FHIR, 2023). However, there are barriers to implementation because of fragmented data, inconsistent coding practices, and interoperability gaps across institutions. Implementing FAIR data principles and adopting standardized vocabularies, such as those from Observational Health Data Sciences and Informatics, can address these issues by enabling consistent data integration, improving usability, and data quality (Pezoulas and Fotiadis, 2024). Data harmonization can, however, be operationally complex and costly due to the need to manage data privacy, consent, and compliance with regulations like the Health Insurance Portability and Accountability Act and EU General Data Protection Regulation (GDPR) (Pezoulas and Fotiadis, 2024).

#### 2.4.2 Building big data platforms founded on robust governance and patient consent

Considering the complexities of data protection and regulation, robust governance is needed to ensure that these frameworks enhance the potential of AI and BDA to address health equity, rather than becoming a significant barrier to progress. In an article by Murdoch (2021), regulation was highlighted as a key issue due to EHR data being among the most private and protected forms of information. The article cautioned that regulation and oversight risk falling behind BDA and emphasized the need for technologically enabled methods of communicating and obtaining patient consent, as well as improved data protection and anonymization (Murdoch, 2021).

An example of the challenges faced when developing and accessing robust EHR data, especially at scale, is the digital transformation of the UK National Health Service (NHS), which is one of the largest employers in the world and received an annual budget exceeding £180 billion in 2023 (NHS Confederation, 2023). Despite its best efforts to go paperless with a digital EHR system, the NHS has failed to meet targets of 100% digitization by 2018–2024, with the last target of March 2026 scrapped and “no set date” now in its place (Clews, 2024; Lovell, 2025). While 90% of NHS trusts have achieved digitization—a notable accomplishment given the organization's scale—the process has been marked by significant delays attributed to difficulties in harmonizing data across primary and secondary care, the slow pace of digital adoption, and lack of fresh thinking and decisive action (Lovell, 2025)—all suggesting potential issues with leadership, strategy, and potentially technical and financial barriers. In a related incident, General Practitioner leaders voiced concerns about patient consent and data governance in an NHS-funded AI model designed to improve predictive healthcare related to COVID-19 vaccinations, ultimately leading to its termination (Colivicchi, 2025). It is therefore clear that, even with significant investment and time, there are issues to overcome regarding the handling and governance of data; however, such challenges are not insurmountable. For example, AI has also been piloted in the NHS to help identify patients who require proactive outreach to address the risk of non-attendance. This approach aims to help patients from marginalized communities get an appointment that works for them, and in doing so improves their outcomes, reduces health inequalities, and lowers costly inefficiencies stemming from missed appointments, all while respecting GDPR and protecting patient data (Deep Medical, 2025). However, as highlighted by Xiao et al. (2025), the potential of AI technologies remains limited by concerns around data privacy, the lack of data-sharing infrastructure, and inconsistent policies, which underscores the need for secure and shared data environments. A privacy-by-design approach has also been recommended by the European Alliance of Associations for Rheumatology (EULAR; formerly the European League Against Rheumatism) and other organizations to ensure that privacy and data protection are embedded at every stage, safeguarding patient information and enabling ethical, compliant, and trustworthy research (Gossec et al., 2020).

### 2.5 Technical capabilities of AI and BDA: challenges and solutions to sources of bias

ML algorithms and AI are being used to facilitate and support earlier diagnosis and optimal treatment in patients with immune diseases by reviewing clinical characteristics and predicting disease and treatment outcomes (Danieli et al., 2024). Considering the known disparities in the diagnosis, treatment, and outcomes of patients with immune disease (Davis et al., 2021), there is potential for AI and BDA to worsen these due to biases within the source data, training data, or the model outputs (Mehrabi et al., 2021). One example, discussed elsewhere, is the use of race adjustment, which requires consideration of risks prior to its application (Vyas et al., 2020).

#### 2.5.1 Ensuring fairness by detecting and addressing blind spots, anomalies, and sources of bias

To avoid exacerbating existing biases, it is essential to engage data scientists and subject-matter experts collaboratively to ensure fairness in big data and AI-powered BDA (Boykin et al., 2021). Part of this is to improve the reliability and accuracy of data through systematic identification of data quality issues using anomaly detection techniques (Gaspar et al., 2011; Churová et al., 2021), along with addressing data blind spots (e.g., biased proxy variables) that perpetuate inequities (Obermeyer et al., 2019).

Detecting systemic and harmful biases in data, models, and outcomes is also critical (Schwartz et al., 2022). This involves understanding and addressing non-random reasons for missing data, implementing methods to identify non-equitable outcomes, and developing decision-support tools to detect biases in patient-generated data, such as EHRs, predictive models, and decision-making processes (Parikh et al., 2019). Fair ML approaches with RWD, including bias mitigation techniques with supervised models (Hardt et al., 2016; Huang et al., 2024), and techniques to detect and correct biases across intersectional subpopulations (Zhang and Neill, 2016; Kearns et al., 2018) should be utilized to mitigate bias. These techniques help identify metrics leading to equitable outcomes, and assess fairness at each step of the algorithm (Suresh and Guttag, 2021; Black et al., 2023).

As an example adapted from the fair ML literature, consider a case where an algorithmic decision-support tool is used to predict the progression of immune disease, and both false positives (incorrectly predicting an increase in severity) and false negatives (incorrectly predicting that severity will not increase) are harmful to patients. Given a concern that a specific protected class defined by a sensitive attribute, such as race or gender (e.g., female patients), are receiving lower-quality predictions, a typical approach (Barocas et al., 2023) is to compare false-positive and false-negative error rates for the protected and non-protected class and identify any statistically significant discrepancies. If the affected subpopulation is not known *a priori*, or there is a concern that the bias may be affecting subgroups defined by multiple data dimensions (e.g., older Black male patients), then techniques such as a bias scan (Zhang and Neill, 2016) can efficiently search across subgroups defined by multiple attributes (race, gender, age, etc.) and identify the subgroups with the most significant error rate imbalances. If biases are detected, approaches for mitigation include adjustment of decision thresholds to balance the error rates (Hardt et al., 2016), resampling or reweighting the data (Kamiran and Calders, 2012), and relearning of the predictive models with additional constraints or penalties to reduce error rate imbalance (Kamishima et al., 2012; Zafar et al., 2017). Alternatively, the detected biases may inform system-wide changes, such as increasing the amount and quality of data collected for population subgroups for whom the algorithmic decision-support tool is performing poorly.

Together, these approaches help to identify and mitigate bias and to develop fairer ML models by balancing error metrics across subgroups. However, even when accounting for these sources of bias, people can misuse data from algorithms in decision-making by discounting algorithmic recommendations in favor of their own judgment, showing tolerance for algorithmic errors, and struggling to evaluate algorithmic performance accurately. They may also be influenced by irrelevant information, trust inaccurate algorithms, and apply algorithms in ways they were not designed for (Green and Chen, 2019), exemplifying the need for increased participation and improved education of subject-matter experts to ensure that AI and BDA are not misused.

### 2.6 Ethical, legal, and data governance models

As outlined in the previous sections, insufficient governance underlies several critical challenges, including weak political and institutional will, limited community engagement, and inadequate technical capabilities in AI and BDA. These governance gaps can lead to inefficiencies, breaches of privacy, misuse of data, and premature termination of AI and BDA projects—ultimately reinforcing existing social inequalities. To address these issues, various governance models comprising structured systems of rules, roles, responsibilities, and processes have been developed to guide decision-making. As shown in Table 3, there are numerous examples addressing ethical, legal, and data governance challenges. However, issues with governance, and indeed BDA, persist largely due to limited awareness, the early-stage nature of many AI-based technologies, and the lack of comprehensive, standalone regulatory frameworks (Palaniappan et al., 2024; Papagiannidis et al., 2025).

**Table 3:** Ethical, legal, and data/algorithmic governance models to address challenges with AI and BDA.

**Domain**	**Challenge/Issue**	**Model/Framework**	**Examples/Tools**
Ethical	Lack of diversity in datasets	Equity-by-design (Lim, 2025)	Inclusive data collection (e.g., NIH All of Us), equity/fairness audits (National Institutes of Health, 2025)
	Opaque AI decision-making	Explainable AI (Chaddad et al., 2023)	SHAP, LIME, ProtoPNet, TCAV
	Inadequate patient control over data use	Dynamic consent models (Kaye et al., 2015)	Platforms allowing real-time consent updates by participants
	Risk of harm from false AI outputs	Risk stratification framework (National Institute of Standards in Technology, 2023)	Continuous safety audits, model recalibration routines
Legal	Data privacy breaches	Data protection laws (Pezoulas and Fotiadis, 2024)	HIPAA (US), GDPR (EU), data minimization principles
	Unclear responsibility for AI errors	Liability and accountability frameworks^*^ (Bovens, 2007; Wieringa, 2020; Cooper et al., 2022)	General accountability and risk assessment frameworks, AI assurance tools, model cards, legal contracts between developers and users
	AI in clinical diagnosis/treatment (regulated space)	Software as a Medical Device regulatory pathways (Palaniappan et al., 2024)	TPLC and GMLP
Data/algorithmic	Sensitive data stored in silos	Federated learning and privacy-preserving AI (Rodríguez-Barroso et al., 2020; Rahman, 2025)	Federated models, differential privacy, homomorphic encryption
	Lack of transparency in dataset origin and use	Data trusts and stewardship agreements (Bartlett, 2024)	Data use agreements, stakeholder governance boards
	Need for auditability and transparency of AI tools	Model fact sheets and data nutrition labels (Stoyanovich and Howe, 2019; Gebru et al., 2021)	Documentation of training data, known biases, limitations
	Integration of diverse data types (EHR, omics, imaging, notes)	Interoperability standards (HL7 FHIR, 2023)	HL7 FHIR, OMOP Common Data Model, FAIR data principles
	Diversity of patient recruitment in immune-focused AI-enhanced clinical trials	Ethical trial design with AI augmentation (Lu et al., 2024)	Bias monitoring, regulatory oversight, subgroup transparency

^*^More clarity is required on liability and accountability frameworks and the need for new laws. A potential AI liability scheme has been proposed by the European Commission (National Telecommunications and Information Administration, 2024).

AI, artificial intelligence; BDA, big data analytics; EHR, electronic health record; FAIR, Findable, Accessible, Interoperable, Reusable; FHIR, Fast Healthcare Interoperability Resources; GDPR, General Data Protection Regulation; GMLP, good machine learning practices; HIPAA, Health Insurance Portability and Accountability Act; HL7, Health Level Seven; LIME, local interpretable model-agnostic explanations; NIH, National Institutes of Health; OMOP, Observational Medical Outcomes Partnership; SHAP, Shapley additive explanations; TCAV, testing with concept activation vectors; TPLC, total product life cycle.

An analysis of global regulatory frameworks revealed that formal regulations are often lacking and soft-law (i.e., voluntary and unenforced) alternatives prevail, such as guidelines, standards, and codes of conduct (Palaniappan et al., 2024). Currently, there is no duty of transparency in the use of healthcare data (Bartlett, 2024) and no established framework for AI liability in the USA (National Institute of Standards in Technology, 2023). In this regulatory void, AI ethics guidelines have proliferated, but are yet to translate into meaningful accountability; vague principles, lack of enforcement mechanisms, and selective implementation continue to undermine their effectiveness (Bartlett, 2024). Ethical commitments often function more as reputational signals, commonly referred to as “ethics washing,” than as governance tools, and the absence of transparency requirements has contributed to the erosion of public trust (Bartlett, 2024).

To address these shortcomings Bartlett (2024) proposed the involvement of a trusted intermediary, or “data steward” to promote public benefit and assume responsibility for the stewardship of health data and the rights of data subjects. A data steward that operates with moral independence from AI developers could manage data on behalf of beneficiaries, enhancing transparency, legitimacy, and public trust (Bartlett, 2024). However, doing so requires a legal entity capable of ensuring accountability: a data trust. A data trust manages data with institutional, legal, and ethical safeguards, while ensuring that stewards remain accountable to beneficiaries. It also helps overcome barriers to the use and sharing of large datasets, offering a more structured and enforceable model of accountability by combining the legal duties of data stewards with participatory oversight (Bartlett, 2024). An example of such an approach is the UK Biobank, a charitable organization that stewards genetic data, and whose board of directors act as charity trustees (i.e., they have oversight and can appoint a data steward) under UK charity law—illustrating how data stewardship can be embedded within a formal governance framework (Hardinges, 2020).

This need for more robust and enforceable governance mechanisms is further illustrated by current regulatory limitations in major jurisdictions. For example, no specific regulatory pathways exist in the USA for AI technologies, which are instead assessed under adapted frameworks designed for traditional medical devices (e.g., Software as a Medical Device) (Palaniappan et al., 2024). This reliance on legacy systems potentially creates oversight gaps, as current regulations may not fully account for the dynamic, adaptive, and autonomous nature of AI (Palaniappan et al., 2024). Without tailored governance frameworks, harm caused by AI systems can persist without redress, particularly given the difficulty of demonstrating such harm due to limited information, inadequate audit trails, and lack of awareness among affected individuals (National Institute of Standards in Technology, 2023). While these challenges remain significant, emerging initiatives such as the draft US-EU voluntary AI code of conduct may offer a path toward greater regulatory convergence and international alignment (Palaniappan et al., 2024).

Importantly, governance deficiencies can also hinder meaningful progress toward health equity. Although many healthcare organizations have expanded their leadership teams to include chief health equity officers or diversity officers, this progress is not always matched by evolution in operating models, governance structures, or budgetary commitments (Aluko et al., 2023). These roles are often under-resourced and under-empowered, limiting their capacity to address systemic disparities (Aluko et al., 2023). Moreover, narrow, business-centric KPIs fail to capture the complex, longitudinal nature of efforts to reduce health disparities, often sidelining equity as a philanthropic afterthought rather than a core strategic goal. A credible health equity strategy needs to be supported by structural change, institutional accountability, and a comprehensive business case that ties equity KPIs to broader organizational success. Embedding these priorities into formal governance and AI/data oversight structures is essential if BDA are to meaningfully contribute to equity rather than exacerbate existing divides.

## 3 Conclusions

Despite substantial investment in health equity, progress on key metrics has been lacking (Zimmerman and Anderson, 2019; Aluko et al., 2023). In immune diseases, existing disparities risk worsening due to the increasing prevalence and burden of disease (Lerner et al., 2015; Cao et al., 2023; Conrad et al., 2023; Global Burden of Disease (GBD) 2019 IMID Collaborators, 2023; Miller, 2023a). While big data, AI, and BDA hold significant potential to address these disparities, past failures and ongoing systemic challenges, such as data quality, governance, and representativeness, must be understood and addressed to deliver meaningful ROI for future investors (Ibrahim et al., 2020; Boykin et al., 2021; Peng et al., 2021; Aluko et al., 2023; Chin et al., 2023; Yerramilli et al., 2024). Big data, AI, and BDA offer transformative potential to address these disparities through earlier diagnosis, tailored treatment, and population-level insights. For example, ML models trained on EHR data have already demonstrated the ability to identify patients in need of further testing, with the potential to accelerate diagnosis and treatment (Forrest et al., 2023) and achieve costs savings with earlier diagnosis (Wylezinski et al., 2019). However, realizing this potential requires confronting and learning from past failures to provide concrete examples evidencing the potential for AI and BDA to address disparities.

A lack of examples evidencing the value and ROI of AI-powered health equity investments in immunology is a key issue and may not be resolved until the central issues discussed in this review—political/institutional will, community engagement, and technical capabilities of AI and BDA—are addressed (Galea and Abdalla, 2023). Underlying these challenges is a lack of robust governance ensuring high-quality, representative data collection through community engagement, standardized data collection practices, and ethical data stewardship, and supports the development and continuous monitoring of meaningful equity-focused KPIs to foster effective communication strategies that demonstrate tangible health equity benefits and secure sustained investment.

Key recommendations from this review, underpinned by a need for robust governance, include, firstly, the need for collaboration among subject-matter experts in health equity, data science/BDA, and immune diseases to develop a communication strategy for key stakeholders to secure engagement and investment. This strategy should include a lexicon of terms and KPIs tailored to demonstrate the benefits of big data, AI, and BDA. Second, investors must ensure funds are spent wisely, with robust governance and performance incentives to prevent wastage and encourage buy-in. Longer-term commitments capable of demonstrating ROI should be prioritized, with input from health equity experts, including improving data and technology infrastructure to understand, target, and track disparities over time. Third, improving the quality of source/training data is a priority to ensure AI and BDA can deliver on health equity. This requires community engagement and input from stakeholders/subject-matter experts. Clear communication about the benefits of representative data and transparent practices is essential to gain community buy-in. For data providers, including healthcare providers and pharmacies, improving data quality necessitates education and accountability at the point of collection and could be underpinned by data collection standards and clear KPIs, including a gold standard for representativeness developed by an interdisciplinary steering committee. These standards should include clauses to ensure disparities are mitigated and data on race, ethnicity, and other social risk factors are consistently collected, with accountability if they are not. Fourth, creating an infrastructure for sharing big data requires harmonizing and standardizing data formats and developing tools to identify data quality issues. This includes adopting FAIR data principles and interoperability standards, such as HL7 FHIR, to facilitate secure, consistent data exchange across systems. Governance frameworks are also needed to emphasize transparency and regulatory compliance, particularly regarding patient data privacy, to overcome barriers to data access. Fair ML approaches should be used to detect and mitigate bias throughout the algorithmic process, ensuring more equitable outcomes.

In summary, investments in big data, AI, and BDA to improve health equity have the potential to address disparities in immune diseases, but success requires a focus on engagement, collaboration, robust governance, meaningful KPIs, continuous monitoring and evaluation, and iterative fairness assessments to ensure a positive ROI.
